# Neonatal azithromycin administration for prevention of infant mortality

**DOI:** 10.1056/EVIDoa2100054

**Published:** 2022-03-17

**Authors:** Catherine E. Oldenburg, Ali Sié, Mamadou Bountogo, Alphonse Zakane, Guillaume Compaoré, Thierry Ouedraogo, Fla Koueta, Elodie Lebas, Jessica Brogdon, Fanice Nyatigo, Thuy Doan, Travis C. Porco, Benjamin F. Arnold, Thomas M. Lietman, NAITRE Study Team

**Affiliations:** 1 Francis I Proctor Foundation, University of California, San Francisco, USA; 2 Department of Ophthalmology, University of California, San Francisco, USA; 3 Department of Epidemiology and Biostatistics, University of California, San Francisco, USA; 4 Centre de Recherche en Santé de Nouna, Burkina Faso; 5 Centre Hospitalier Universitaire Pédiatrique Charles-de-Gaulle, Ouagadougou, Burkina Faso

## Abstract

**Background.:**

Biannual mass azithromycin administration reduces all-cause childhood mortality in some sub-Saharan African settings, with the largest effects in children aged 1-5 months. Azithromycin has not been distributed to children <1 month due to risk of infantile hypertrophic pyloric stenosis (IHPS).

**Methods.:**

This 1:1 placebo-controlled trial, randomized neonates aged 8-27 days to a single oral dose of azithromycin (20 mg/kg) or equivalent volume of placebo in 5 regions of Burkina Faso during 2019 and 2020. The primary outcome was all-cause mortality at 6 months of age. Infants were evaluated at 21 days after treatment and at 3 and 6 months of age for vital status; family and provider surveillance for IHPS continued throughout.

**Results.:**

Of 21,832 enrolled neonates, 10,898 were allocated to azithromycin and 10,934 to placebo. At 6 months of age, 92 infants had died, 42 (0.44%) in the azithromycin group and 50 (0.52%) in the placebo group (hazard ratio 0.85, 95% confidence interval 0.56 to 1.28, *P*=0.46). A single IHPS case was detected, which was in the azithromycin arm. Serious adverse events, including death and hospitalization within 28 days of treatment, occurred in 0.27% of infants in the azithromycin group and 0.14% in the placebo group, for an absolute risk difference 0.14 percentage points, 95% confidence interval 0.01 to 0.26.

**Conclusions.:**

Overall mortality was lower than anticipated when the trial was designed, thus limiting its power. The available data do not support the routine use of azithromycin for prevention of mortality in neonates in sub-Saharan African settings similar to the one in which this trial was conducted.

**Trial registration.:**

ClinicalTrials.gov NCT03682653

## INTRODUCTION

Although substantial declines in under-five mortality have been documented worldwide, infant (<1 year) and neonatal (<1 month) mortality remain persistently high in some geographic regions.^1,2^ Most childhood mortality after the first week of age is infectious.^3,4^ Biannual mass azithromycin administration has been shown to reduce all-cause childhood mortality in some settings in sub-Saharan Africa.^5^ Among children aged 1 through 5 months, biannual mass azithromycin distribution reduced mortality approximately 25% compared to placebo.^5^ Children in this age group had the highest mortality rates, suggesting that infants may benefit the most from any implementation of azithromycin for prevention of childhood mortality.^6^

Mass distribution of azithromycin is currently limited to children >1 month of age due the risk of infantile hypertrophic pyloric stenosis (IHPS). IHPS is a rare condition that requires timely, i.e., within days to weeks, identification and can be fatal in the absence of surgery.^7^ Observational studies in high resource settings have suggested an association between macrolide use in early infancy and increased risk of IHPS.^8,9^ However, these studies are limited by confounding by indication, as infants receiving macrolides may be systematically different than those receiving a different antibiotic class or no antibiotic. Randomized controlled trials of azithromycin use during the neonatal period have been limited to very low birthweight neonates for prevention of bronchopulmonary dysplasia.^10,11^ These trials have been underpowered to detect any effect of azithromycin on IHPS.

Given results from MORDOR^5^ that found the greatest benefit of azithromycin for prevention of mortality in infants <6 months of age and due to lack of safety data for neonatal azithromycin, we conducted a randomized placebo-controlled trial to evaluate the efficacy and safety of single dose neonatal azithromycin for prevention of infant mortality. We hypothesized that single dose oral azithromycin administered to neonates would reduce all-cause mortality by 6 months of age and that there would be no effect of azithromycin on development of IHPS.

## METHODS

### Study overview.

The *Nouveux-nés et Azithromycine: une Innovation dans le Traitement des Enfants* (NAITRE) trial was a 1:1 randomized placebo-controlled evaluating the efficacy of a single oral 20 mg/kg dose of azithromycin compared to matching placebo administered to neonates aged 8 to 27 days for prevention of infant mortality at 6 months of age (see Supplemental Material for full trial protocol).^12^ The study was reviewed and approved by the Comité d’Ethique pour la Recherche en Santé (National Research Ethics Committee) in Ouagadougou, Burkina Faso (Protocol #2018-10-123) and the Institutional Review Board at the University of California, San Francisco (Protocol #18-25027). Written informed consent was obtained from the caregiver of each enrolled child.

### Study setting.

The study was conducted in 44 *Centres de Santé et de Promotion Sociale* et Centres Médicaux in 5 regions of Burkina Faso (Figure S1). These are primary healthcare facilities that offer basic preventative and curative care, such as antenatal care and vaccination visits. CSPSs were located in 9 regions in 5 districts of Burkina Faso (Centre, Centre Ouest, Boucle du Mouhoun, Hauts-Bassins, and Cascade). Study sites were selected to be within 4 hours of a facility capable of performing pyloromyotomy for IHPS (Centre Hospitalier Universitaire Pédiatrique Charles de Gaulle in Ouagadougou or Centre Hospitalier Universitaire Souro Sanou in Bobo Dioulasso). Burkina Faso experiences highly seasonal rainfall and a co-occurring high malaria season. Children aged 3-59 months living in the study area are eligible to receive seasonal malaria chemoprevention monthly from July through October.

### Recruitment.

Participants were recruited from participating health facilities via facility-based births and on bacille Calmette-Guerin vaccination days (held weekly at each facility). Study staff contacted women who gave birth in participating facilities to inform them about the study and how to participate. On vaccination days, caregivers attending the vaccination clinic were informed about the study.

### Eligibility criteria.

Neonates were eligible if they were between 8 and 27 days of age, weighed at least 2500 g at the time of enrollment (due to hypothesized increased risk of IHPS among underweight infants^7^), had no known allergies to azalides or macrolides, were able to feed orally (to take the study medication), and had no neonatal jaundice based on clinical signs such as scleral icterus and jaundice (potentially indicating hepatic insufficiency). Neonates who were too young or too small at their first evaluation could return for a second evaluation and possible inclusion in the trial if they then met all eligibility criteria. Because neonates in the first week of life may be more vulnerable, the Data and Safety Monitoring Committee recommended on evaluation of the study design, that all infants less than 8 days of age be excluded from the trial.

### Intervention.

Participants were randomized in a 1:1 fashion to a single oral 20 mg/kg dose of azithromycin or matching placebo. Neonates were weighed on a standard infant scale (ADE M112600 U Scale) for weight-based dosing and the study’s electronic data capture application automatically calculated the volume equivalent to a 20 mg/kg dose of azithromycin. Matching placebo was indistinguishable in appearance, taste, and smell to azithromycin and had the same composition except for the active ingredient. Study treatment was delivered orally by syringe and all study treatments were directly observed by the study nurse.

### Randomization.

Participants were individually randomized after enrollment and baseline assessment in a 1:1 fashion to azithromycin or matching placebo. Due to the large sample size and expectation that baseline characteristics would be well balanced, the trial used simple unrestricted randomization.^13^ The randomization sequence was generated by the trial’s unmasked data team using R (The R Foundation for Statistical Programming, Vienna, Austria).

### Masking and allocation concealment.

Masking was achieved via utilization of the placebo. Study medication bottles were labeled identically except for a randomly assigned letter (e.g., ”A”, “B”, etc) that corresponded to azithromycin or placebo. Only the study’s unmasked data team were aware of which letters corresponded to azithromycin or placebo. After informed consent and enrollment, each participant was assigned a study identification number. To facilitate allocation concealment, the letter associated with the participant’s study identification number was only revealed by the study’s electronic data capture application after all baseline procedures were complete. Study participants, caregivers, staff, and investigators were masked to treatment allocation.

### Study visits.

Participants were evaluated at enrollment (baseline), 21 days (up to 28 days) from enrollment, and at 3 and 6 months (with a pre-specified window for the visit of plus or minus 42 days) of age. The primary endpoint was 6 months of age.

### Primary outcome.

The primary pre-specified endpoint was all-cause mortality by 6 months of age. At each study visit, study staff recorded the child’s vital status (alive, died, moved, or unknown). A child contributed towards the prespecified primary mortality endpoint if they were recorded as having died between enrollment and their 6-month visit, allowing for a +/− 6 week vital status ascertainment window (age 141 to 225 days).

### Pre-specified secondary outcomes.

Pre-specified secondary outcomes included mortality before 28 days of age, death and/or hospitalization by 6 months of age, hospitalization at each study time point (21 days and 3 and 6 months of age), and sick-child visits at primary healthcare facilities at each study time point. Hospitalization and healthcare visits were collected via caregiver report during each study visit. Caregivers were asked to specify reasons for hospitalization and primary healthcare visits, which excluded well-child visits such as vaccination appointments.

### Adverse event screening.

IHPS screening occurred via active screening at the 21-day visit, via carereport, and evaluation of all children who presented to a study facility for a non-planned visit. Caregivers were asked to report to the study team should their children exhibit symptoms of IHPS. Children with symptoms suspicious for IHPS, including projectile vomiting and vomiting after every feed, were referred for a diagnostic ultrasound. Full details of the adverse event screening protocol are in the “Supplemental Methods” section of the Supplemental Appendix.

### Trial oversight.

The trial was overseen by a Data and Safety Monitoring Committee (See the “List of Investigators” in the Supplemental Appendix) comprising of experts in biostatistics, epidemiology, pediatrics, pediatric infectious disease, and bioethics. The Committee met prior to the study’s start to review and approve the protocol, and annually during the course of the study. Any serious adverse events deemed potentially related to study treatment were sent to the Committee for review as soon as possible after event recognition.

### Sample size.

Sample size calculations were based on the primary endpoint, probability of mortality by 6 months of age. We assumed a mortality probability of 35 per 1000 live births in the placebo arm based on region-level estimates from the Institute for Health Metrics and Evaluation, which estimated mortality from birth to 12 months of age^2^, and loss to follow-up of 10%. Under these assumptions, a sample size of 10,856 per arm (*N*=21,712 total) would yield approximately 80% power to detect a 20% decrease in 6-month mortality among children receiving azithromycin compared to placebo.

### Interim analysis.

A single interim analysis for efficacy at an alpha of 0.001 was pre-specified when the first one-third of the planned study population (*N*=7,238) reached their primary endpoint or at the end of the first full year of enrollment, whichever occurred first. Full methods for the interim analysis are in the “Supplemental Methods” section of the Supplemental Appendix.

### Statistical methods.

Unless otherwise indicated, all analyses considered a two-sided alpha of 0.05 statistically significant. All analyses were conducted in R (The R Foundation for Statistical Computing). The primary prespecified analysis was binomial regression with a complementary log-log link, with treatment group as the sole predictor to estimate the relative hazard of mortality between groups. P-values were estimated using a two-sided permutation test of the log hazard ratio (10,000 iterations). Pre-specified subgroup analyses for the primary outcome included age of enrollment by week (2, 3, or 4 weeks of age), sex (male or female), season of enrollment (*rainy*, defined as June through October, or *dry*), region (Centre, Boucle du Mouhoun, Cascade, Centre Ouest, or Hauts-Bassins), and urbanicity (urban, peri-urban, or rural, where urban is a town with running water and electricity, peri-urban the outskirts of a town and without running water or electricity, and rural outside of a town and without running water or electricity). A non-prespecified sensitivity analysis included all children with vital status measurements, regardless of whether the visit was in the pre-specified follow-up visit window.

The prespecified analysis for IHPS was a one-sided 90% confidence interval of the estimated relative risk of IHPS among infants randomized to azithromycin compared to placebo. Adverse events were analyzed as the proportion of individuals in each arm reporting any adverse event and each adverse event individually, with corresponding risk differences and 95% confidence intervals. Secondary outcomes included hospitalization and/or death and primary healthcare visits (overall and for specific reasons: malaria, pneumonia, diarrhea, and fever in the absence of a separate diagnosis). Secondary outcome analyses used the same methods as the mortality analysis. In addition, total healthcare visits were analyzed using negative binomial regression.

## RESULTS

### Enrollment

**Figure 1.**
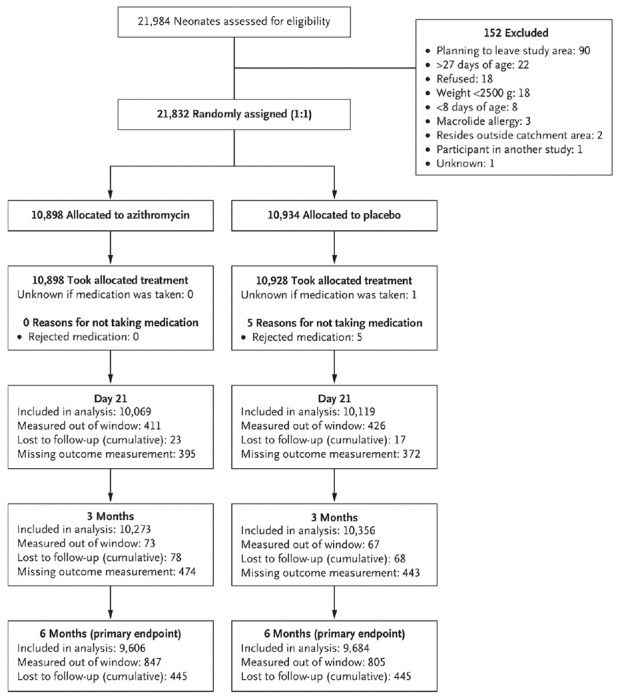
Screening, randomization, and follow-up of participants

Among 21,832 neonates enrolled in the study, 10,898 were allocated to azithromycin and 10,934 to placebo (Figure 1). Enrollment occurred from April 2019 through December 2020 and the last follow-up visit was completed in July 2021. Five neonates allocated to placebo did not receive their allocated study medication due to not being receptive to taking the study medication. At enrollment, participants were a median of 11 days old in each group and 49.7% were female (Table 1). Median birthweight was 3000 g in each group and participants had a median body weight of 3300 g at enrollment. At 6 months, 9,606 (88%) infants in the azithromycin arm and 9,684 (89%) in the placebo arm were measured in the pre-specified 6-month visit window and included in the primary analysis. An additional 847 infants in the azithromycin arm and 805 in the placebo arm were measured out of window and included in a sensitivity analysis (96% of enrolled infants in each arm included in sensitivity analysis). Baseline characteristics did not differ between infants who were and were not lost to follow-up and measured out of the pre-specified window (Table S1). The Data Safety and Monitoring Committee recommended continuation of the trial upon review of the interim analysis results (Table S2).

### Primary Outcome

By 6 months of age, 92 infants died, 42 (42/9606 or 0.44%) in the azithromycin group and 50 (50/9684 or 0.52%) in the placebo group (Table 2; hazard ratio, HR, 0.85, 95% confidence interval, CI, 0.56 to 1.28, *P* = 0.46). Sensitivity analysis including all children regardless of measurement window did not change results (Table S3). There was no evidence of a difference in mortality in infants receiving azithromycin compared to placebo in any pre-specified or non-prespecified subgroup (Table 2; Table S4).

### Other outcomes

There was no evidence of a difference in any pre-specified secondary endpoints, including death and/or hospitalization at 6 months (Table 3). Deaths before 28 days of age were uncommon likely due to the overlapping enrollment period (8-27 days of age) and because neonates were only enrolled from day 8 onward, thus excluding any deaths that would have happened in the first week of age. Before 28 days of age, 9/10,485 (0.09%) of neonates in the azithromycin group and 6/10,547 (0.06%) of neonates in the placebo group died. By six months of age, more than 1/3 of children had sought care for a sick-child reason at a clinic, but there was no difference in useage by treatment group (azithromycin: 36.1%, placebo: 36.6%, HR 0.98, 95% CI 0.94 to 1.03). Results were similar when tabulating total number of clinic visits by arm (Table S5).

### Adverse Events

Serious adverse events (death and/or hospitalization) through 28 days from treatment administration occurred in 44 participants; they were more common in the azithromycin group compared to placebo (Table 4). A total of 29 (0.27%) serious adverse events were reported in the azithromycin group compared to 14 (0.14%) in the placebo group (RD, risk difference, 0.14 percentage points, 95% CI 0.01 to 0.26). Causes of these serious adverse events are listed in Table S4. A single case of IHPS was reported in a male infant who was treated at 10 days of age who was in the azithromycin group. Four infants received a diagnostic ultrasound for IHPS, 2 in the azithromycin group and 2 in the placebo group (Table S5). There was no difference in overall non-serious adverse events by treatment group (azithromycin: 8.11%, placebo: 8.39%, RD −0.3 percentage points, 95% CI −1.18 to 0.62). Vomiting was more frequently reported in children receiving azithromycin versus placebo (RD 0.41 percentage points, 95% CI 0.10 to 0.72) and fever was less common in children receiving azithromycin versus placebo (RD −0.60 percentage points, 95% CI −1.16 to −0.04%).

## DISCUSSION

In this randomized controlled trial of neonates aged 8-27 days of age randomized to placebo or single dose azithromycin versus placebo, we were unable to demonstrate a difference in all-cause mortality at 6 months of age. Although the effect was consistent with a 15% reduction in mortality, similar to what has been demonstrated when azithromycin is administered to children aged 1-59 months^5^, confidence intervals were wide, indicating a large degree of uncertainty in the estimate. Based on the mortality rate observed, the study did not have power needed to detect a reduction in mortality in infants receiving azithromycin compared to placebo. If the mortality rates we observed were preserved, a sample size of almost 250,000 children would have been required for 80% power to observe a difference that reached statistical significance. In the MORDOR study, biannual mass azithromycin distribution to entire communities led to a 14% reduction in mortality in infants 1-59 month of age, with a 25% reduction in mortality among infants aged 1-5 months in Niger, the study site with the highest childhood mortality rates.^5^ In the present study, selection of enrollment sites required proximity to national hospitals with capability for pyloromyotomy in case a child was diagnosed with IHPS and children who weighed <2500 g were excluded due to concerns for increased risk of IHPS. As a result, we observed considerably lower infant mortality rates than the MORDOR study and in the general population in Burkina Faso (Table S7). Azithromycin for infant mortality in settings with low mortality rates may not be beneficial for further reducing infant mortality.

A single case of IHPS was detected by the study’s active surveillance system during the course of the trial. IHPS has been linked to early macrolide use in observational studies in the United States and Europe. This study was designed specifically to ensure early detection and intervention if IHPS symptoms were observed in a study participant. In the United States and Europe, the incidence of IHPS is approximately 2 per 1,000 live births in the absence of macrolide exposure.^7,14,15^ In sub-Saharan Africa the incidence is thought to be much lower, although the data on IHPS is derived from case series of children admitted to hospitals with confirmed IHPS^16,17^, rather than data derived from cohort studies, precluding estimates of incidence. The present study suggests that IHPS is very rare in the study population (approximately 0.05 cases per 1,000 live births). The single case of IHPS in this study was in the azithromycin arm, however the rarity of this outcome precludes any conclusions related to causality related to the use of azithromycin and development of IHPS. Overall, vomiting was rarely reported after treatment, and was reported less often than in previous studies among older children.^18,19^

Neonates receiving azithromycin had a higher risk of a serious adverse event during the 28-day period post treatment compared to those receiving placebo. The absolute risk of serious adverse events was less than 5 per thousand over the 6 month observation period. Serious adverse events were most commonly neonatal death or hospitalization. All serious adverse events were evaluated by the study’s medical monitors and most were judged to not be possibly related to study participation, with the exception of the IHPS case and two sudden neonatal deaths that occurred within 24 hours of treatment administration. However, autopsies were not available for either case and thus a cause of death was not available, precluding formal causality assessment. All three serious adverse events judged to be possibly related to study participation were in the azithromycin arm. These results suggest that early azithromycin may lead to a small increased risk in mortality and morbidity within the first few weeks of administration among neonates, which may have balanced any potential benefit several weeks after treatment.

This study had several limitations. The event rate for the primary outcome, all-cause mortality by 6 months of age, was about 9 fold lower than expected when the sample size was estimated; this led a substantial loss of statistical power with wide confidence intervals for the primary outcome comparison. Reasons for the lower than anticipated mortality rate are likely due to exclusion of children <8 days of age and <2500 g at enrollment due to concerns related to increased risk of IHPS and because children had to be enrolled in facilities close to hospitals with pediatric surgical capability, potentially selecting for higher socioeconomic status locations. Azithromycin distribution has been recommended in settings with infant mortality of >60 per 1000 live births, considerably higher than that observed in the present study. Results from MORDOR demonstrated some evidence of heterogeneity by background childhood mortality rate, with most of the overall effect of azithromycin for prevention of childhood mortality in Niger, where childhood mortality rates were much higher than in the study sites in Malawi and Tanzania.^5,20^ The present trial cannot determine if neonatal azithromycin distribution would reduce infant mortality in a higher mortality population. We did not collect data on seasonal malaria chemoprevention, although infants aged 3-6 months during the rainy season (June through October) would have been eligible for up to 4 distributions of seasonal malaria chemoprevention. Given the randomized nature of the trial and that all children aged 3-59 months regardless of morbidity are eligible for seasonal malaria chemoprevention, we do not anticipate an imbalance in treatment arms in children who did and did not receive seasonal malaria chemoprevention. We are unable to comment on whether affects efficacy of azithromycin in this study. We did not collect data on childhood vaccination status, but similarly do not anticipate that vaccination status differed by arm. Finally, we did not collect laboratory samples for assessment of microbiome outcomes. The infant microbiome undergoes rapid maturation during the first few months of age, and azithromycin has been shown to alter the composition of the gut microbiome in young children.^21,22^ Early azithromycin may cause changes to the infant microbiome that could affect longer-term outcomes.

In conclusion, a single oral dose of azithromycin administered to neonates aged 8 to 27 days did not significantly reduce the risk of all-cause mortality by 6 months of age compared to placebo. Although serious adverse events were rare, they occurred more frequently in the azithromycin arm compared to the placebo arm. These results do not support the routine use of azithromycin for prevention of mortality in neonates in low mortality sub-Saharan African settings.

## Supplementary Material

Supp Material

## Figures and Tables

**Table 1.** Baseline characteristics by treatment group

	Azithromycin (N=10898)	Placebo (N=10934)	Overall (N=21832)
**Age (days)**			
Median (IQR)	11 (9 to 15)	11 (9 to 14)	11 (9 to 14)

**Sex**			
Female	5413 (49.7%)	5431 (49.7%)	10844 (49.7%)
Male	5485 (50.3%)	5503 (50.3%)	10988 (50.3%)

**Region**			
Centre	919 (8.4%)	951 (8.7%)	1870 (8.6%)
Boucle du Mouhoun	1299 (11.9%)	1329 (12.2%)	2628 (12.0%)
Cascade	2009 (18.4%)	1977 (18.1%)	3986 (18.3%)
Centre Ouest	1217 (11.2%)	1211 (11.1%)	2428 (11.1%)
Hauts-Bassins	5454 (50.0%)	5465 (50.0%)	10919 (50.0%)

**Season of enrollment**			
Rainy (June – October)	5217 (47.9%)	5295 (48.4%)	10512 (48.1%)
Dry (November – May)	5681 (52.1%)	5639 (51.6%	11320 (51.9%)

**Birthweight (g)**			
Median (IQR)	3000 (2700 to 3250)	3000 (2700 to 3260)	3000 (2700 to 3250)

**Weight at enrollment (g)**			
Median (IQR)	3300 (2980 to 3620)	3300 (2990 to 3620)	3300 (2990 to 3620)

**Length at enrollment (cm)**			
Median (IQR)	50.4 (49.3 to 51.9)	50.5 (49.3 to 52.0)	50.5 (49.3 to 51.9)

**Pregnancy type**			
Singleton	10702 (98.2%)	10753 (98.3%)	21455 (98.3%)
Multiple	195 (1.8%)	177 (1.6%)	372 (1.7%)

**Initiation of breastfeeding**			
Immediate	10320 (94.7%)	10341 (94.6%)	20661 (94.6%)
Delayed	566 (5.2%)	574 (5.2%)	1140 (5.2%)
Not breastfeeding	11 (0.1%)	15 (0.1%)	26 (0.1%)

**Current breastfeeding**			
Exclusive	10859 (99.6%)	10901 (99.7%)	21760 (99.7%)
Not exclusive	27 (0.2%)	14 (0.1%)	41 (0.2%)
Does not breastfeed	11 (0.1%)	15 (0.1%)	26 (0.1%)

**Mother’s age**			
Median (IQR)	25 (21 to 30)	25 (21 to 30)	25 (21 to 30)

**Mother’s education**			
None	5910 (54.2%)	6029 (55.1%)	11939 (54.7%)
Primary	1990 (18.3%)	1978 (18.1%)	3968 (18.2%)
Secondary or above	2997 (27.5%)	2923 (26.7%)	5920 (27.1%)

**No. of antenatal visits**			
Median (IQR)	4 (3 to 5)	4 (3 to 5)	4 (3 to 5)

Abbreviation: IQR, interquartile range

**Table 2.** Primary outcome (6-month mortality) and subgroup analyses for the primary outcome

	Azithromycin			Placebo				

6-month mortality	N	n	%	N	n	%	HR (95% CI)	P-value*
All participants	9606	42	0.44%	9684	50	0.52%	0.85 (0.56 to 1.28)	0.46

**Age at enrollment**								
8 to 14 days	7207	31	0.43%	7347	37	0.50%	0.85 (0.53 to 1.38)	
15 to 21 days	1634	5	0.31%	1562	8	0.51%	0.60 (0.20 to 1.82)	
22 to 28 days	765	6	0.78%	774	5	0.65%	1.22 (0.37 to 3.98)	

**Child’s sex**								
Female	4748	20	0.42%	4786	22	0.46%	0.92 (0.50 to 1.68)	
Male	4858	22	0.45%	4898	28	0.57%	0.79 (0.45 to 1.38)	

**Season of enrollment**								
Rainy (June-October)	4667	16	0.34%	4757	24	0.50%	0.68 (0.36 to 1.28)	
Dry (November-May)	4939	26	0.53%	4927	26	0.53%	1.00 (0.58 to 1.72)	

**Region**								
Centre	874	4	0.46%	904	5	0.55%	0.83 (0.22 to 3.08)	
Boucle du Mouhoun	1009	2	0.20%	1055	6	0.57%	0.35 (0.07 to 1.72)	
Cascade	1792	14	0.78%	1766	13	0.74%	1.06 (0.50 to 2.26)	
Centre Ouest	1062	4	0.38%	1050	4	0.38%	0.99 (0.25 to 3.95)	
Hauts-Bassins	4869	18	0.37%	4908	22	0.45%	0.82 (0.44 to 1.54)	

**Urbanicity** **								
Urban	7239	30	0.41%	7333	32	0.44%	0.95 (0.58 to 1.56)	
Rural	1698	9	0.53%	1653	14	0.85%	0.62 (0.27 to 1.44)	
Peri-urban	663	3	0.45%	688	4	0.58%	0.78 (0.17 to 3.48)	

Abbreviations: N, number measured; n, number of participants who died; CI, confidence interval

* Permutation P-value (10,000 replicates);

** The urbanicity of 15 children in the trial is unknown

**Table 3.** Secondary outcomes by treatment group

	Azithromycin			Placebo			

	N	n	%	N	n	%	HR (95% CI)
Death before 28 days of age	10485	9	0.09%	10547	6	0.06%	1.51 (0.54 to 4.24)

3-month mortality	10273	23	0.22%	10356	20	0.19%	1.16 (0.64 to 2.11)

Death and/or hospitalization, 6 months	9567	112	1.17%	9642	110	1.14%	1.03 (0.79 to 1.33)

**Hospitalization**							

21 days	10069	14	0.14%	10119	7	0.07%	2.01 (0.81 to 4.98)
3 months	10261	27	0.26%	10354	16	0.15%	1.70 (0.92 to 3.16)
6 months	9584	107	1.12%	9668	103	1.07%	1.05 (0.80 to 1.37)

**Any clinic visit**							

21 days	10071	561	5.57%	10122	570	5.63%	0.99 (0.88 to 1.11)
3 months	10286	1419	13.80%	10377	1379	13.29%	1.04 (0.97 to 1.12)
6 months	9620	3476	36.13%	9702	3554	36.63%	0.98 (0.94 to 1.03)

**Reason for clinic visit, 6 months**							

Malaria	9620	478	4.97%	9702	468	4.82%	1.03 (0.91 to 1.17)
Pneumonia	9620	1489	15.48%	9702	1441	14.85%	1.05 (0.97 to 1.12)
Diarrhea	9620	641	6.66%	9702	670	6.91%	0.96 (0.86 to 1.07)
Fever^1^	9620	603	6.27%	9702	636	6.56%	0.95 (0.85 to 1.07)

Abbreviations: N, number measured; n, number of participants with secondary outcome; CI, confidence interval;

1 Fever without another diagnosis

**Table 4.** Adverse events within 28 days of treatment by treatment group

Serious Adverse Event	Azithromycin (N = 10898)	Placebo (N = 10934)	RD (95% CI) Percentage Points
	n (%)	n (%)	
Any serious adverse event	29 (0.27%)	15 (0.14%)	0.14 (0.01 to 0.26)
Infantile hypertrophic pyloric stenosis (IHPS)	1 (0.01%)	0 (0.00%)	0.01 (0.00 to 0.03)
Mortality within 28 days of treatment	16 (0.15%)	8 (0.07%)	0.08 (−0.02 to 0.17)
Hospitalization within 28 days of treatment	14 (0.13%)	7 (0.06%)	0.07 (−0.02 to 0.15)
Non-serious Adverse Event	Azithromycin (N = 7089)	Placebo (N = 7138)	RD (95% CI) Percentage Points
	n (%)	n (%)	
Any non-serious adverse event	575 (8.11%)	599 (8.39%)	−0.28 (−1.18 to 0.62)
Vomiting (Any)	78 (1.10%)	49 (0.69%)	0.41 (0.10 to 0.72)
Vomiting after every feed	22 (0.31%)	9 (0.13%)	0.18 (0.03 to 0.34)
Projectile vomiting	3 (0.04%)	0 (0.00%)	0.04 (0.00 to 0.10)
Diarrhea	51 (0.72%)	61 (0.85%)	−0.14 (−0.43 to 0.16)
Fever	193 (2.72%)	237 (3.32%)	−0.60 (−1.16 to −0.04)
Abdominal Pain	209 (2.95%)	171 (2.40%)	0.55 (0.02 to 1.08)
Rash	70 (0.99%)	86 (1.20%)	−0.22 (−0.56 to 0.12)
Constipation	82 (1.16%)	111 (1.56%)	−0.40 (−0.78 to −0.02)

Abbreviations: N, number measured; n, number of participants with adverse event; RD, risk difference (azithromycin - placebo), in percentage points; CI, confidence interval
