# Supplementary material for: Neonatal azithromycin administration for prevention of infant mortality
Source: NEJM Evid. Author manuscript. (PMC9172759; doi:10.1056/EVIDoa2100054)
Supplement: Supp Material [file EVIDoa2100054_Supplement.pdf]

Supplemental Appendix for "Neonatal azithromycin administration for prevention of infant mortality"

TABLE OF CONTENTS

LIST OF INVESTIGATORS 2

AUTHOR CONTRIBUTIONS 3

SUPPLEMENTAL METHODS 4

ADVERSE EVENT SCREENING PROTOCOL. 4
INTERIM ANALYSIS METHODS. 4
PROTOCOL CHANGES. 5

FIGURE S1. MAP OF STUDY ENROLLMENT SITES 6

TABLE S1. BASELINE CHARACTERISTICS BY RETAINED OR NOT RETAINED IN THE PRIMARY ANALYSIS 7

TABLE S2. INTERIM ANALYSIS RESULTS FOR SAFETY AND EFFICACY 8

TABLE S3. SENSITIVITY ANALYSIS FOR THE PRIMARY OUTCOME 9

TABLE S4. NON-PRESPECIFIED SUBGROUP ANALYSES FOR THE PRIMARY OUTCOME 10

TABLE S5. CAUSES OF SERIOUS ADVERSE EVENTS 11

TABLE S6. SCREENING AND REFERRAL FOR INFANTILE HYPERTROPHIC PYLORIC STENOSIS 12

TABLE S7. COUNT OUTCOMES FOR NUMBER OF CLINIC VISITS AMONG NEONATES RANDOMIZED TO AZITHROMYCIN OR PLACEBO. 13

TABLE S8. BACKGROUND INFORMATION ON THE POPULATION AFFECTED BY ALL-CAUSE INFANT MORTALITY BETWEEN 7 DAYS AND 6 MONTHS OF AGE IN BURKINA FASO 14

List of Investigators

Clinical Centers

Centre de Recherche en Santé de Nouna, Nouna, Burkina Faso
Ali Sié (Principal Investigator), Mamadou Bountogo, Mamadou Ouattara, Boubacar Coulibaly, Cheikh Bagagnan, Alphonse Zakane, Clarisse Dah, Mariam Seynou, Thierry Andiyam Ouedraogo, Guillaume Compaoré, Valentin Boudo, Adama Compaoré, Fla Koueta, Eric Nebie, Idrissa Kouanda, Constantin Sow, and Ourohire Millogo

Centre Hospitalier Universitaire Pédiatrique Charles De Gaulle, Ouagadougou, Burkina Faso
Fla Koueta, A.M. Napon

Centre Hospitalier Souro Sanou, Bobo Dioulasso, Burkina Faso
Z. Nikièma

Supervisors, Burkina Faso
Jacques Kalmogo, Paul Yamdare, Fatimata Lengane, Adama Deme, Ibrahim Sebrene, Elisee Sawadogo, and Dramane Kiemdé

Healthcare Workers, Burkina Faso
Issa Ouohono, Fatoumata Sankara, Afoussatou Soule, Patricia Dala, Aminata Yameogo, Gabin Timboué, Malika Zoure, Abel Coulibaly, Aïda Drabo, Marietou Barry, Richard Simboro, OUsseni Tingueri, Pélagie Yameogo, Georgette Nikiema, Françoises Nakelsé, Safiata Semdé, Sandrine Nignan, Antoinette Sawadogo, Madiana Yao, Siéfenbo Hema, Nouratou Soma, Aboloubouye Koné, Aïssata Soman, Aboubacar Sorgho, Mousso Yameogo, Mariam Sessouma, Rachidatou Ouedraogo, Boureima M'Ganame, Arouna Zongo, Koko Tioyé, Hadjara Ouohara, Minata Yaro, Agathe Ousse, Samuel Karama, Rihanata Zabre, Clarisse Sanou, Kady Camara, Arouna Coulibaly, Pascaline Dao, and Rissanta Kabore

Committees

Data and Safety Monitoring Committee:
Allen Hightower (Chair), Amza Abdou, Miriam Laufer, Jacqueline Glover, and Wafaie Fawzi

Resource Centers

Trial Coordinating Center, F.I. Proctor Foundation, University of California, San Francisco, California, USA:
Thomas M. Lietman, Benjamin F Arnold, Catherine E. Oldenburg, Travis C. Porco, Thuy Doan, Jeremy D. Keenan, Kieran O'Brien, Elodie Lebas, Fanice Nyatigo, Huiyu Hu, Jessica Brogdon, Ying Lin, William Godwin

Heidelberg University, Heidelberg Germany:
Till Bärnighausen

Author Contributions

The study was designed by CEO, AS, MB, EL, TD, TCP, and TML. Data collection and supervision was done by CEO, AS, MB, AZ, GC, TO, FK, EL, JB, FN, and TML. CEO, TCP, and BFA vouch for the data and the analysis. CEO wrote the first draft of the paper and all authors critically reviewed the paper. All authors decided to submit for publication.

Supplemental Methods

Adverse event screening protocol. Screening for infantile hypertrophic pyloric stenosis (IHPS) was conducted via active screening and caregiver report. During informed consent, all caregivers were informed of the risk of IHPS and signs to look for. They were asked to inform the study staff should their child experience projectile vomiting or vomiting after every feed. At the 21-day post-treatment visit, caregivers were asked a series of questions about vomiting. Caregivers of children with persistent projectile vomiting and/or who were vomiting after every feed were referred for an ultrasound. Note that children could be referred for an ultrasound outside of the active screening program, and children whose caregivers reported projectile vomiting that had resolved were not necessarily referred. Children who presented to a study facility for a non-planned study visit were screened for IHPS.

An ultrasound was considered positive for IHPS if the pyloric muscle thickness (diameter of a single muscular wall on a transverse image) was >4 mm, the length of the pylorus was >15 mm, or pylorus thickness and length were below these criteria, but no food could pass. The infant was transferred to a pediatric surgical facility in Ouagadougou or Bobo Dioulasso for further workup and pyloromyotomy if indicated. Prior to transfer, infants were assessed for electrolyte disturbance and dehydration and were stabilized. Any infant receiving surgery for IHPS was followed at 1 week and 4 weeks after the procedure to assess the child's vital status and monitor recovery. In addition to the active screening protocol, caregivers were educated about the risk of IHPS during the informed consent procedure and were asked to contact study staff immediately in the event their child developed projectile vomiting or if they were otherwise concerned about IHPS or another adverse event.

All serious adverse events, including IHPS, as well as hospitalization and deaths within 28 days of treatment, were reported to the study's medical monitors within 24 hours of the study team becoming aware of them. If the medical monitors deemed the serious adverse event to be possibly related to study treatment, they were reported to the Data and Safety Monitoring Committee (DSMC) within 24 hours.

In addition to IHPS and other serious adverse events, caregivers were asked at the 21-day visit about other non-serious adverse events that their child had experienced since treatment. These including diarrhea, fever, abdominal pain, rash, and constipation. Non-serious adverse events were reported overall and by specific category. Non-serious adverse event data were collected through August 2020 (for approximately two-thirds of participants).

Interim analysis methods. A single interim analysis for efficacy at an alpha of 0.001 was pre-specified when the first one-third of the planned study population (N=7,238) reached their primary endpoint or at the end of the first full year of enrollment, whichever occurred first. The one-third enrollment goal was achieved in February 2020 and the interim analysis was completed in October 2020. The interim analysis additionally included an analysis of futility using simulation, with a stipulation to discontinue the trial at the discretion of the DSMC if conditional power dropped below 10%. Interim analyses for safety included tabulation of IHPS cases by study group, and the pre-specified interim analysis plan stipulated that while statistical tests would be reported, the decision to discontinue for safety would not be made solely on the basis of statistical considerations. Finally, all other adverse events were summarized overall and by study group, including the risk difference for adverse events by arm and corresponding 95% confidence intervals.

Protocol changes. The trial's original protocol stipulated 3 weekly follow-up visits for adverse events at days 7, 14, and 21 after treatment. Due to the low incidence rate of IHPS and reports of projectile vomiting, the adverse event screening was changed in July 2020 from 3 weekly visits to a single adverse event screening at 21 days after treatment. At the time the change was implemented, 14,591 participants had been enrolled. This change was reviewed and approved by the DSMC and in the Institutional Review Boards in the United States and Burkina Faso. During implementation of the change, an error was introduced in the study's mobile data application that affected branching logic for adverse event questions was introduced, which meant that children who had a 21-day visit after this change was made were not actively screened for adverse events. Data were collected for these children regarding clinic visits and hospitalizations, and serious adverse events were collected as usual and reported to the DSMC. After the error was discovered, the study team traced children enrolled after the change for whom the study had no collected vital status to determine whether any of the children could have had IHPS. No suspected cases of IHPS were detected through this follow-up. For analytic purposes, an infant was considered to have experienced an adverse event if they had experienced one on any of the 3 follow-up visits prior to the change in protocol. No other protocol changes were implemented during the course of the trial.

Figure S1. Map of study enrollment sites

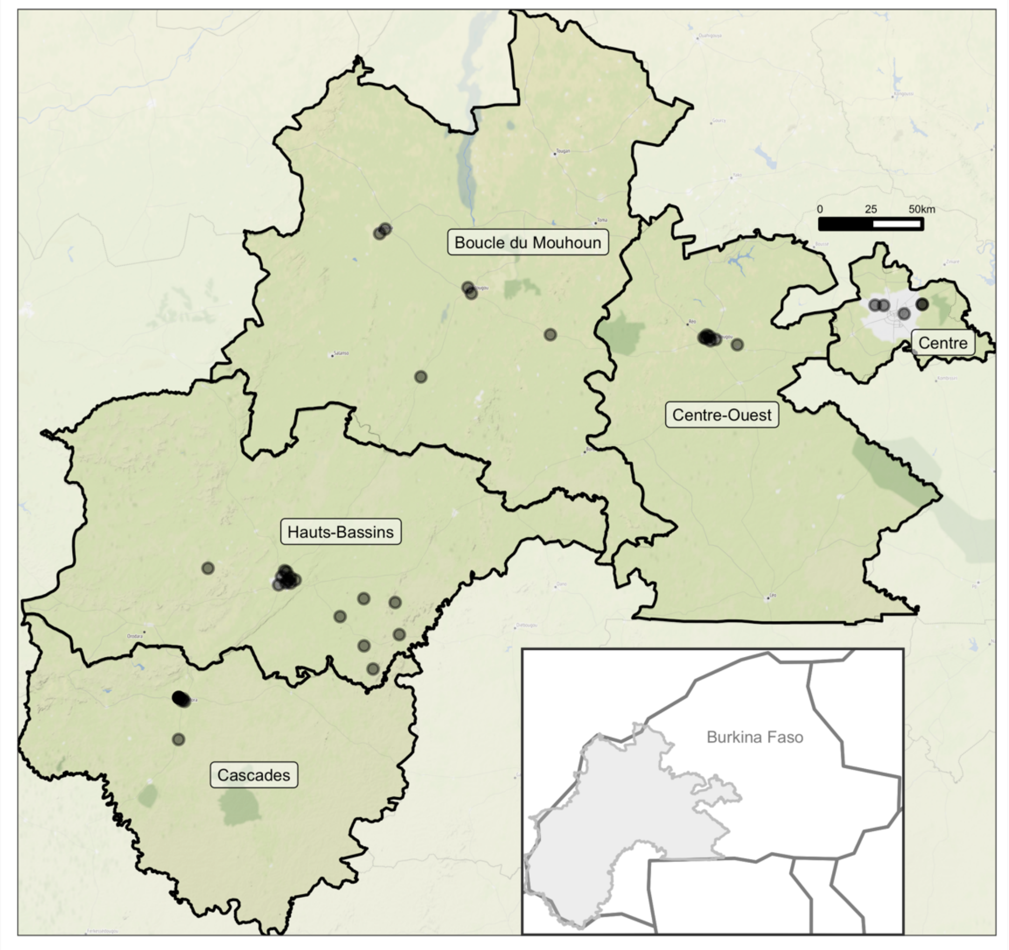

Table S1. Baseline characteristics by retained or not retained in the primary analysis (6-month mortality among children measured in the pre-specified window)

	Retained (N=19290)	Not retained (N=2542)	Overall (N=21832)
**Age (days)**			
Median (IQR)	11 (9 to 14)	11 (9 to 15)	11 (9 to 14)
**Sex**			
Female	9534 (49.4%)	1310 (51.5%)	10844 (49.7%)
Male	9756 (50.6%)	1232 (48.5%)	10988 (50.3%)
**Region**			
Centre	1778 (9.2%)	92 (3.6%)	1870 (8.6%)
Boucle du Mouhoun	2064 (10.7%)	564 (22.2%)	2628 (12.0%)
Cascade	3558 (18.4%)	428 (16.8%)	3986 (18.3%)
Centre Ouest	2112 (10.9%)	316 (12.4%)	2428 (11.1%)
Hauts-Bassins	9777 (50.7%)	1142 (44.9%)	10919 (50.0%)
**Season of enrollment**			
Rainy (June – October)	9424 (48.9%)	1088 (42.8%)	10512 (48.1%)
Dry (November – May)	9866 (51.1%)	1454 (57.2%)	11320 (51.9%)
**Birthweight (g)**			
Median (IQR)	3000 (2700 to 3260)	3000 (2700 to 3200)	3000 (2700 to 3250)
**Weight at enrollment (g)**			
Median (IQR)	3300 (2990 to 3620)	3290 (2980 to 3630)	3300 (2990 to 3620)
**Length at enrollment (cm)**			
Median (IQR)	50.5 (49.3 to 52.0)	50.2 (49.2 to 51.6)	50.5 (49.3 to 51.9)
**Pregnancy type**			
Singleton	18933 (98.1%)	2522 (99.2%)	21455 (98.3%)
Multiple	352 (1.8%)	20 (0.8%)	372 (1.7%)
**Initiation of breastfeeding**			
Immediate	18317 (95.0%)	2344 (92.2%)	20661 (94.6%)
Delayed	946 (4.9%)	194 (7.6%)	1140 (5.2%)
Not breastfeeding	22 (0.1%)	4 (0.2%)	26 (0.1%)
**Current breastfeeding**			
Exclusive	19222 (99.6%)	2538 (99.8%)	21760 (99.7%)
Not exclusive	41 (0.2%)	0 (0%)	41 (0.2%)
Not breastfeeding	22 (0.1%)	4 (0.2%)	26 (0.1%)
**Mother's age**	19222 (99.6%)	2538 (99.8%)	21760 (99.7%)
Median (IQR)	25 (21 to 30)	25 (20 to 30)	25 (21 to 30)
**Mother's education**			
None	10517 (54.5%)	1422 (55.9%)	11939 (54.7%)
Primary	3547 (18.4%)	421 (16.6%)	3968 (18.2%)
Secondary or above	5221 (27.1%)	699 (27.5%)	5920 (27.1%)
**No. of antenatal visits**			
Median (IQR)	4 (3 to 5)	4 (3 to 5)	4 (3 to 5)

Abbreviation: IQR, interquartile range

Table S2. Interim analysis results for safety and efficacy (1/3 of total anticipated study population)

	**Azithromycin**	**Placebo**		
**Safety**	**N (%)**	**N (%)**	**RD (95% CI)**	
Fever	193 (2.1%)	237 (2.6%)	-0.49 (-0.93 to -0.05)	
Abdominal pain	209 (2.3%)	171 (1.9%)	0.41 (0 to 0.83)	
Constipation	82 (0.9%)	111 (1.2%)	-0.32 (-0.62 to -0.02)	
Rash	70 (0.8%)	86 (0.9%)	-0.18 (-0.44 to 0.09)	
Diarrhea	51 (0.6%)	61 (0.7%)	-0.11 (-0.34 to 0.12)	
Vomiting	78 (0.9%)	49 (0.5%)	0.32 (0.08 to 0.56)	
Hospitalized and/or died	95 (1.0%)	90 (1.0%)		
**Efficacy**	**N**	**N**	**HR (95% CI)**	***P*-value***
6-month mortality	29 (0.6%)	27 (0.6%)	1.08 (0.64 to 1.82)	0.77

*Permutation *P*-value based on 10,000 replicates

Table S3. Sensitivity analysis for the primary outcome (mortality at 6 months of age) including all children with vital status information regardless of whether the child was measured in the pre-specified measurement window for the primary analysis. The hazards ratio (HR) was estimated with a binomial regression model with a complementary log-log link with treatment group as the sole predictor. The *P*-value was estimated used a two-sided permutation test of the log hazard ratio with 10,000 iterations.

	Azithromycin			Placebo				
**6-month mortality**	**N**	**n**	**%**	**N**	**n**	**%**	**HR (95% CI)**	**P-value***
All children regardless of window	10453	42	0.4%	10489	50	0.5%	0.84 (0.56 to 1.27)	0.42

Table S4. Non-prespecified subgroup analyses for the primary outcome

	Azithromycin			Placebo			
	**N**	**n**	**%**	**N**	**n**	**%**	**HR (95% CI)**
**Breastfeeding**							
Exclusive	9570	42	0.4%	9652	50	0.5%	0.85 (0.56 to 1.28)
Not exclusive	8	0	0%	14	0	0%	NA
Not breastfeeding	27	0	0%	14	0	0%	NA
**Weight at enrollment**							
<2800 g	1334	20	1.5%	1309	17	1.3%	1.16 (0.60 to 2.21)
≥2800 g	8272	22	0.3%	8374	33	0.4%	0.67 (0.39 to 1.16)

Abbreviations: N, number measured; n, number of participants who died; CI, confidence interval

Table S5. Causes of serious adverse events (death and/or hospitalization within 28 days of study treatment) by treatment group.

	**Azithromycin**		**Placebo**
**Reason for SAE**	**Death**	**Hospitalization**	**Death**
Abdominal bloating	1	0	0
Accident	1	0	0
Bleeding	1	0	0
Failure to feed	1	0	0
Fever	1	0	2
Fever and anemia	1	0	0
Fever and sunken fontanel	1	0	0
Malnutrition	1	0	0
Respiratory distress	1	1	0
Unknown	3	0	2
Diarrhea, vomiting (not IHPS)	0	1	0
IHPS	0	1	0
Fever and hypoglycemia	0	0	1
Loss of consciousness	0	0	1
Pain on rib palpation	0	0	1
**Total***	**12**	**3**	**7**

*Includes only participants for whom a serious adverse event was reported in real time and included a narrative of the serious adverse event. Cause determination is per description in the narrative form of the serious adverse event.

Table S6. Screening and referral for infantile hypertrophic pyloric stenosis

	**Azithromycin**	**Placebo**
Projectile vomiting identified during active screening	3	0
Referred to healthcare facility during active screening	1	1
Referred for an ultrasound	2	2
Diagnosed with IHPS	1	0

Table S7. Count outcomes for number of clinic visits among neonates randomized to azithromycin or placebo.

	Azithromycin			Placebo			
**Count Outcome**	**N**	**n**	**mean**	**N**	**n**	**mean**	**Means Ratio (95% CI)**
Clinic, Total- 21 days	561	642	1.14	570	657	1.15	0.98 (0.88 to 1.09)
Clinic, Total- 3 months	1392	1821	1.31	1354	1785	1.32	1.03 (0.96 to 1.10)
Clinic, Total- 6 months	3428	5930	1.73	3513	6032	1.72	0.99 (0.96 to 1.03)

Table S8. Background information on the population affected by all-cause infant mortality between 7 days and 6 months of age in Burkina Faso

Category	
Condition under investigation	All-cause infant mortality by 6 months of age
Special considerations related to	
Age	Infant mortality rates typically decrease as children get older. After the first week of life, most causes of infant mortality in Burkina Faso are infectious. The infant mortality rate in Burkina Faso (including deaths from birth through 12 months of age) was approximately 53 per 1000 live births in 2019 according to UNICEF. The majority of infant mortality occurs by 6 months of age.
Race or ethnic group	Infant mortality in Burkina Faso primarily affects Black Africans (native Burkinabè).
Gender	Infant mortality (from birth through 12 months of age) is higher in Burkina Faso in male compared to female infants (57 deaths per 1000 live births in male infants versus 48 deaths per 1000 live births in female infants in 2019 per UNICEF).
Other considerations	Infant mortality is higher in rural compared to urban settings, where socioeconomic status is generally lower and there is reduced access to healthcare. Infant mortality is higher in undernourished babies and those born low birthweight.
Overall representativeness of this trial	Participants in this trial were 50% male and 50% female, as expected. The infant's biological sex was ascertained by asking the caregiver if the infant was male or female. Infants were most commonly enrolled during the 2nd week of age, and median age at enrollment was 11 days, so the age distribution at enrollment skewed younger than the general population. All participants were Black. Participants were more often urban compared to rural-dwelling due to the need to enroll participants near facilities with pediatric surgical capacity. Participants were less often low birthweight and underweight compared with the general population, and thus likely to have lower mortality rates than the general population of Burkina Faso.

Data on trends in infant and childhood mortality in Burkina Faso was based on UNICEF data from 2019.
